# Growth Mechanism of Strain-Dependent Morphological Change in PEDOT:PSS Films

**DOI:** 10.1038/srep25332

**Published:** 2016-04-29

**Authors:** Yoo-Yong Lee, Gwang Mook Choi, Seung-Min Lim, Ju-Young Cho, In-Suk Choi, Ki Tae Nam, Young-Chang Joo

**Affiliations:** 1 Department of Materials Science and Engineering, Seoul National University, Seoul 151-744, Korea, Republic of Korea; 2 High Temperature Energy Materials Research Center, Korea Institute of Science and Technology (KIST), Seoul, 136-791, Republic of Korea; 3 Institute of Physics (IA), RWTH Aachen University, Aachen, 52056, Germany

## Abstract

Understanding the mechanism of the strain-dependent conductivity change in polymers in stretched conditions is important. We observed a strain-induced growth of the conductive regions of PEDOT:PSS films, induced by a coalescence of conductive PEDOT-rich cores. This growth due to coalescence leads to a gradual decrease in the electrical resistivity up to 95%, independent of the thickness of the PEDOT:PSS films. The primary mechanism for the evolution of the PEDOT-rich cores proceeds by the cores growing larger as they consuming relatively smaller cores. This process is caused by a strain-induced local rearrangement of PEDOT segments in the vicinity of PSS shells around the cores and also changes the chemical environment in PEDOT, induced by the electron-withdrawing effects around the PEDOT chains. The strain-induced growth mechanism is beneficial to understanding the phenomenon of polymeric chain rearrangement in mechanical deformation and to modulating the electrical conductivity for practical applications.

The resistivity of materials generally increases under large mechanical strain and does not recover after the deformation due to the generation of defects (buckles and cracks)^1^,^2^,^3^ and geometrical plasticity^4^,^5^. However, within the elastic strain region, strain-dependent resistivity can increase or decrease depending on the intrinsic properties of the material. For example, the resistivity of n-doped silicon decreases^6^,^7^, whereas that of p-doped silicon^6^,^8^,^9^ and metals^10^ increases due to the change in the atomic lattice, which alters the electronic band structures. Such piezoresistive properties have been used in many applications such as pressure sensors^11^, strain gauges^12^, and accelerometers^13^. With the increasing demand for stretchable and flexible devices, the development of materials that exhibit reliable deformation-induced resistivity changes, even under large amounts of deformation, has become necessary^5^,^14^,^15^,^16^,^17^,^18^, and the underlying mechanism of the resistivity change under such large deformations must be understood.

As a flexible and stretchable electrode, the soft polymeric PEDOT:PSS (poly(3,4-ethylenedioxythiophene):polystyrene sulfonate) conductor is widely applicable in electronic devices^19^,^20^,^21^,^22^,^23^. However, its electrical behaviors in response to mechanical deformation have not been clearly elucidated. The relative change in the resistance of this material under 2% strain, known as the gauge factor, was previously reported to be 5.2^24^ and 17.8^25^. These values indicate a positive increase in resistivity with strain. However, the opposite change in resistivity has also been observed when the applied strain was increased up to 10%: the measured resistivity gradually decreased with the increasing strain^26^,^27^. Above 10% strain, the resistivity change cannot be observed due to the generation of defects (buckles and cracks) and the change in resistivity could occur in a complex manner. Considering all the previous data, the range of values over which the resistivity changes and the mechanisms that have been proposed for this change are inconsistent. To understand the strain-dependent resistivity change of PEDOT:PSS, this study focused on the morphological change of the PEDOT-rich core, which is known to be a major determinant of the intrinsic conductivity of the material.

The morphology of PEDOT:PSS has a particular core-shell structure composed of conductive and tangled PEDOT-rich cores surrounded by shells with excess PSS chains^28^,^29^. PEDOT has very short and lightweight segments compared with polymeric PSS chains^29^. The main role of PSS is counter-ion for PEDOT conductive polymer. To transport mobile electrical carriers in PEODT, PEDOT should be in a positively charged state that has unpaired π electrons which is the doped state. Simultaneously, for the thin film formation, it requires an additive to be in a water-soluble form of the PEDOT due to the hydrophobicity of PEDOT. In this respect, PSS is the most suitable additive that meets the requirements for PEDOT. The PSS is a highly water-soluble and a negatively charged state in an aqueous solution. By combining PSS with PEDOT, the positive charge of PEDOT can be maintained and dispersed in a solution to form a polymeric dispersion. In the cores, positively charged PEDOT is electrostatically bound to the negatively charged PSS chains via Coulomb interactions. The excess PSS chains that are not bonded to PEDOT surround the cores with a shell thickness of 5–6 nm^28^. Because the cores have a much higher conductivity than the PSS shells, their conductivity is predominantly determined by the size of the cores and the phase distribution of the cores and the shells in the films. The size and film resistivity of the PEDOT-rich cores are highly correlated. For example, when increasing the PSS content, the core diameter and the shell thickness decrease and increase, respectively, and the intrinsic conductivity decreases due to the reduction of the diameter of the conductive cores. The morphology can be controlled by the mixing ratio of PEDOT-to-PSS^30^, UV irradiation^31^,^32^, and thermal treatment^33^, which cause the change in the conductivity. In a more direct way, mechanical deformations induce morphological changes and can modulate the electrical properties over a wide range. However, the detailed underlying mechanisms of the external strain-induced morphological change of PEDOT:PSS have not been studied previously. In this regard, we investigated the origin of morphological changes in PEDOT-rich cores under large tensile deformation and the correlation of the resistivity change induced by the resultant morphological changes.

In other conducting polymers, such as polyacetylene^34^, polyaniline^35^ and P3HT^36^, the resistivity always decreases during stretching. This behavior is primarily due to the directional alignment of polymer chains along direction of the mechanical stress and the subsequent conformational change from entangled chains to linear chains. This phenomenon is more interesting for PEDOT:PSS, which consists of two types of polymers: a single PSS chain with adherent PEDOT segments. Uniquely, the conductive part of PEDOT is oligomeric and bonded to a relatively long PSS chain, and not all PSS chains contain PEDOT segments. Thus, the mechanism of the decrease in strain-induced resistivity differs from that of single-component conducting polymers.

In this study, we investigated the strain-dependent resistivity change through an in-depth understanding of the morphological evolution of PEDOT:PSS during stretching above 60% strain. The resistivity decreased by following an identical decreasing path that was independent of the PEDOT:PSS film thickness, and the decrease in resistivity can be explained by the growth of the conductive PEDOT-rich cores with applied tensile strain. The conductive cores gradually grow larger by consuming relatively smaller cores as the strain of the films increases. We further elucidated the growth mechanism caused by the local rearrangement of PEDOT segments in the vicinity of PSS shells. The resultant rearrangement leads to changes in the chemical environment in the cores, such as changes to the bonding energy and binding energy on PEDOT. We successfully examined the growth mechanism of the conductive cores induced by the local rearrangement of PEDOT segments, which directly modulates the electrical, morphological, and chemical properties that are dependent on the strain.

## Results

### Resistivity change dependent upon the PEDOT:PSS thickness under tensile strain

**Figure 1:**
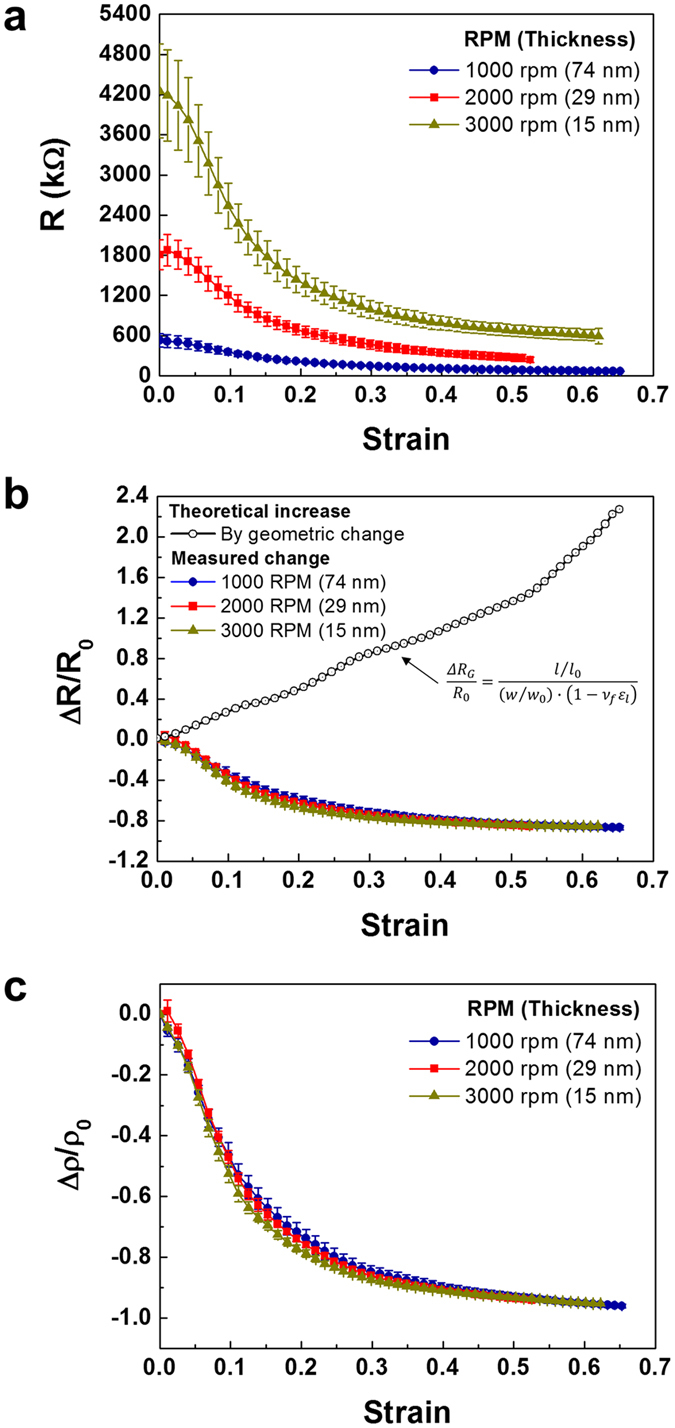
Tensile strain induced decrease in the resistivity independent on the thickness. (**a**) The resistance change of PEDOT:PSS films with tensile strain according to the thickness of the films. (**b**) Relative change in the resistance (ΔR/R_0_) with the strain. Gray circle indicated theoretical increase in resistance by geometrical change. (**c**) Relative change in resistivity (Δρ/ρ_0_) as a function of tensile strain.

To investigate the tensile strain-responsive electrical behaviors of PEDOT:PSS films, we observed the change in resistance under tensile deformation that is dependent on the thickness of the PEDOT:PSS films, which were spin-coated on PI substrates at different revolutions per minute (Figure S1). The strain was measured by a calculation of distance change between two markers on the PEDOT:PSS film with an optical microscope. The displacement rate was 0.5 mm/sec until the rupture of the film. The indication accuracy of strain is within ±1% indicated value. The resistance change as a function of tensile strain is shown in Fig. 1a for the different thicknesses of the PEDOT:PSS films. The electrical resistance was measured *in-situ* upon stretching above 60% strain, and the tensile deformation was terminated after mechanical rupture of the films.

As shown in Fig. 1a, the initial resistance of the films increased due to the decrease in thickness. Notably, when the PEDOT:PSS films were stretched, their resistance gradually decreased, even when elongated above 60% strain. During mechanical stretching of PEDOT:PSS films of different thicknesses, the decrease in resistance is enabled by the stretch, regardless of the thickness. To quantify the decrease in resistance of the PEDOT:PSS films with respect to tensile strain, we plotted the relative change in resistance (ΔR/R_0_) as a function of the strain (Fig. 1b). Interestingly, despite the large difference in initial resistance depending on the PEDOT:PSS film thickness, the relative resistance change decreased along the same path as the strain on the films increased. The change in resistance gradually reduced with increasing strain and reached saturation, which is 86% of the decrease in the resistance change for all the PEDOT:PSS films. The relative change in resistance includes an increase in theoretical resistance induced by a change in the geometrical shape of the films. We also plotted the theoretical increase curve in Fig. 1b based on optical measurements of the change in length (*l/l*_*0*_) and width (*w/w*_*0*_) with increasing strain and a calculation of the thickness change (1 − *v*_*f* _*ε*_*l*_) assumed following Poisson’s compression. In spite of a large increase in the relative resistance due to the geometrical change, the measured resistance change decreased by following the same path, independent of the PEDOT:PSS film thickness. This phenomenon has not been observed previously.

To investigate the intrinsic electrical response with tensile strain, the electrical resistivity change was obtained by excluding the theoretical increase in resistance from the measured relative change in resistance. By rearranging the measured change in resistance (∆*R/R*_*0*_) and the theoretical increase in resistance (∆*R*_*G*_*/R*_*0*_), the relative change in electrical resistivity (∆*ρ/ρ*_*0*_) can be obtained. The detailed calculation procedure is described in the Supporting Information. The equation is as follows:


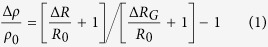


We plotted the relative resistivity change as a function of tensile strain for the PEDOT:PSS films of different thicknesses, as shown in Fig. 1c. The resistivity change gradually decreased by as much as 95% with the strain of the films, and interestingly followed the same decreasing path of resistivity as the strain, independent of the thickness. The decreasing behaviors were only induced by the mechanical strain on the PEDOT:PSS films. The mechanical deformation affected the morphological changes in PEDOT:PSS to reduce the resistivity of the PEDOT:PSS films, independent of the thickness. Hence, we investigated the mechanism controlling the decrease in resistivity by elucidating the morphological changes in PEDOT:PSS with increasing strain.

### Evolution of the PEDOT:PSS morphology under tensile deformation

**Figure 2:**
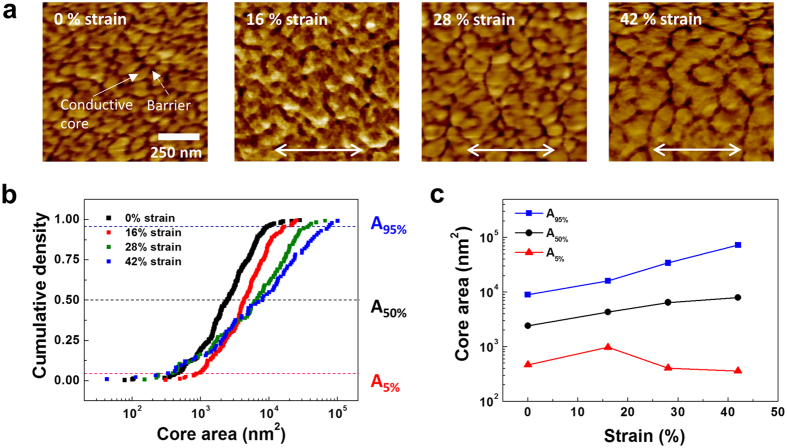
Morphological change of PEDOT:PSS according to the applied tensile strain. (**a**) AFM phase images of PEDOT:PSS films at each strain denoted in the images. Bright and dark fields represent the PEDOT-rich cores and the PSS shells, respectively. The arrow indicates the stretching direction, and all the images were obtained by using tapping-mode AFM at a scale of 1 × 1 μm^2^. (**b**) Distribution of cumulative density as a function of PEDOT-rich core area by using log-normal distribution function. (**c**) The change in the area PEDOT-rich cores at 5%, 50%, and 95% of cumulative density as function of tensile strain.

A mechanical strain directly affects the morphological changes in PEDOT:PSS and leads to a decrease in the electrical resistivity. The PEDOT:PSS morphology consists of tangled and conductive PEDOT-rich cores surrounded by insulating shells composed of excess PSS chains. We examined the PEDOT:PSS morphology after tensile deformation by observing AFM phase images, which are presented in Fig. 2a. The bright and dark fields in the images indicate PEDOT-rich cores and PSS shells, respectively. The size of the conductive cores mainly determines the electrical conductivity of the PEDOT:PSS films^37^,^38^. When the tensile strain increased, the overall size of the cores increased, independent of the direction of stretching. Because of the equivalent growth of the PEDOT-rich cores independent of the tensile direction, the resistivity at the direction perpendicular to the tensile direction decreased with the strain as well (Figure S2).

To easily differentiate between the cores and shells with increasing strain, the AFM phase images were reproduced with binary contrast (black and white) in Photoshop (Figure S3), which clearly revealed large increases in the conductive cores and a reduced number of cores and shells per unit length (1 μm) with increasing strain of the films. The increase in the conductive area and the decrease in insulated barriers enhance the mobile charge transport. As previously confirmed, the equivalent decrease in resistivity occurred independent of the thickness of PEDOT:PSS, indicating that the growth of the conductive cores was the main mechanism for the improvement of electrical conductivity, regardless of the thickness.

The evolution of the PEDOT-rich cores is significantly influenced by an applied strain. To quantify the increase of the cores with increasing strain, we measured each area of the cores at the strain of the PEDOT:PSS films, corresponding to the black regions in the reproduced images (Figure S3). The size of an individual core can be obtained from the image analysis software (Scion Image Analyzer). The distribution of the core area was plotted as the log-normal distribution, which is commonly used to present the distribution function of living tissue domains or the grain size of thin films. The cumulative equation of the log-normal distribution is as follows.


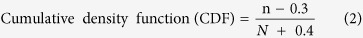


where n is each core number and N is the total number of cores. Using the size of the cores and their total number, we can obtain the cumulative density of the cores. The cumulative distribution as a function of the core area was plotted at each strain of the PEDOT:PSS films, as shown in Fig. 2b. With increasing strain, the curves shifted to the right, and the slope decreased, which indicates an increase in the median size and standard deviation of the cores. It means that non-uniform growth of the cores occurred with increasing strain.

We observed the evolution according to the initial PEDOT-rich core size at the sub-5% (A_5%_), median (A_50%_), and top-95% (A_95%_) levels. The area change of each core was plotted as a function of the tensile strain (Fig. 2c). The cores evolve with the strain, regardless of their initial area. The median core size was 0.23, 0.42, 0.51, and 0.61 × 10^−2^ μm at a strain of 0%, 16%, 28%, and 42%, respectively. The median size increased by 85%, 119%, and 164% with increasing strain compared to the initial core size. This large increase in the conductive region is a major cause of the enhancement in electrical conductivity. Such a large core growth cannot only be explained by mechanical elongation due to tensile stress. Interestingly, the non-uniform behavior of the core growth was dependent on the initial size as the strain increased in the films. Initially, large cores (A_95%_) gradually became larger as the strain increased. Conversely, the area occupied by small cores (A_5%_) steadily decreased with increasing strain, indicating that the PEDOT-rich cores evolve through the consumption of small cores by large cores with increasing strain. The overall increase in the conductive area is mainly caused by the growth of large-sized cores according to the tensile strain.

When we closely observed the morphological change of PEDOT:PSS with increasing strain, boundaries between the PEDOT-rich cores gradually disappeared and combined with each other. The growth of the cores occurred through coalescence or agglomeration among the cores, and the median size increased by 85%, 119%, and 164% with increasing strain compared to the initial core size. Such a large core growth cannot solely be explained by mechanical elongation due to tensile stress. For example, at a strain of 42%, the increase in the surface area of the cores induced only by mechanical deformation was 16%, which is much smaller than the actual core growth (164%). Hence, the coalescence among the PEDOT-rich cores induced by the strain is the main cause of the core growth. Our questions concern how the increase in the conductive core area responds to the applied strain and what occurs inside of the cores. We believe that a local rearrangement of PEDOT chains occurs in the vicinity of the PSS shells with increasing strain.

### Change in the chemical environmental induced by PEDOT rearrangement

A local rearrangement of the PEDOT segments changes the chemical environment of PEDOT:PSS. To investigate this change, spectroscopic analyses were conducted using X-ray photoelectron spectroscopy (XPS) and Fourier-transform infrared (FT-IR) spectroscopy. The detailed chemical inter-chain interaction involves PEDOT chains being bonded to PSS chains via Coulomb interactions. The PSS is a dopant material for PEDOT and donates a proton with a sulfonate group. However, there are many PSS chains that are not bonded to PEDOT chains but are distributed around the PEDOT-rich cores. The local rearrangement of the PEDOT in the PSS shells generates the change in the bonding interactions between PEDOT and the PSS chains.

**Figure 3:**
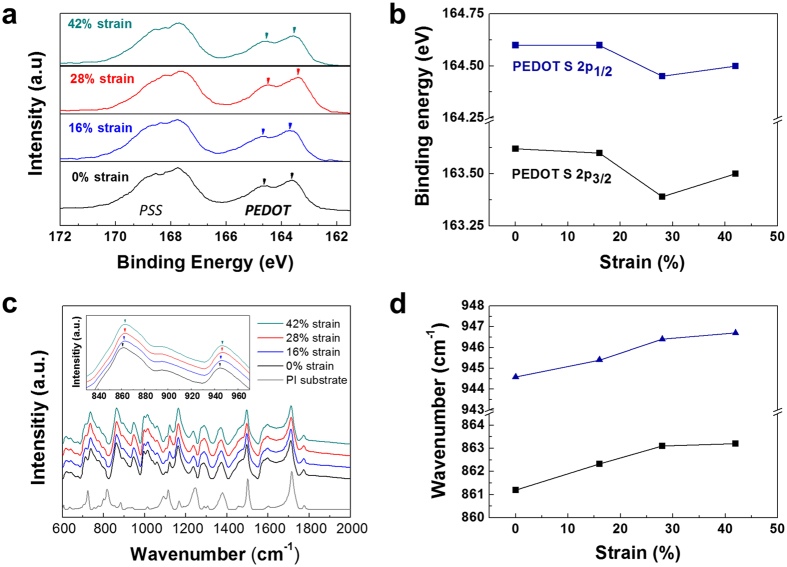
Spectroscopic analysis on the change in bonding energy with tensile strain. (**a**) XPS spectra of S 2p for PEDOT:PSS films as a function of applied strain (0, 16, 28, and 42%). (**b**)The change in binding energy of S 2p in PEDOT according to the strain. (**c**) FT-IR absorption spectra for PEDOT:PSS films with the tensile strain and PI substrates. The inset shows the peak shift of C-S bonding. (**d**) The change in carbon-sulfur (C-S) bonding energy in PEDOT chain as a function of the strain.

To examine the electron binding energy change of PEDOT:PSS with increasing strain, the sulfur (S 2p) XPS spectrum was analyzed. Figure 3a shows the S 2p binding energy change as a function of the deformation strain. The S 2p line contains a spin-split doublet consisting of S 2p1/2 and S 2p3/2. In the spectra of the PEDOT:PSS films, the lower and higher binding energy peaks correspond to the sulfur atoms in PEDOT and PSS, respectively. The binding energy of the sulfur in PSS is higher than that of the sulfur in PEDOT because the PSS sulfur is strongly positive due to the presence of three oxygen atoms in the sulfonate group around the sulfur atom^39^,^40^. The distribution of the binding energy of PEDOT is more spread out due to the delocalization of the positive charge of PEDOT over several adjacent rings.

When we observed the change in the binding energy with increasing strain, the energy distribution shifted to the lower energy side, which means that the required energy for detaching the electron decreased due to the applied strain. The change in binding energy of the sulfur in PEDOT as a function of the deformation strain is shown in Fig. 3b and reveals a decrease in the binding energy as the strain increased. The binding energy is shifted by 0.3 eV above a strain of 16%. Combined with the previous results, the PEDOT rearrangement into the shells directly affects not only the increase in the core area but also the decrease in binding energy of the sulfur in PEDOT. Considering the reasons for the decrease, two main factors influence the reduction in the atoms’ binding energies. First, the decrease in oxidation number decreases the binding energy due to increased screening of the bound electron from the ion core. Second, the local chemical environment also shifts the binding energy. The electronegativity change surrounding the atoms can decrease the energy required to detach electrons due to the change in the electron density of the atoms. The binding energy of sulfur in PSS-Na reportedly increased by 0.4 eV compared with that in PSS-H due to an increased electronegative effect surrounding the sulfur in PSS^39^. Here, the latter affects the decrease in electron binding energy of the sulfur in PEDOT. The local rearrangement of PEDOT into the shell changes the electrons, which increases the amount of PSS chains around PEDOT, by drawing the outer electron of sulfur in PEDOT to the side of the PSS chains, induced by the increase in electronegativity. Thus, less energy is required to detach the electrons from the sulfur in PEDOT.

The bonding energy between constituent atoms in PEDOT:PSS could also change with the strain because of the rearrangement of PEDOT segments. To verify the bonding energy change, FT-IR spectra were recorded for the PI substrate and the PEDOT:PSS films after deformation up to 0, 16, 28, and 42% strain (Fig. 3c). In the spectra of the films, the peak positions corresponding to the bonding energies are listed in Table S1^41^,^42^,^43^. In particular, to examine the bonding energy change in the sulfur in PEDOT, we observed the peak position at approximately 862 and 944 cm^−1^ corresponding to the carbon-sulfur (C-S) bonding energy with the deformation strain as representative peaks. These peaks shifted to higher bonding energies, as shown in the inset of Fig. 3c. We also show the C-S bonding energy change in PEDOT as a function of the strain, which reflects the increased bonding energy with increased strain (Fig. 3d). This result was caused by the electron drawing effect on the sulfur in PEDOT due to the increased amount of PSS chains around the PEDOT segments. The sulfur appeared to be more negative in the C-S bonding, which caused a small vibrational energy increase in the bonding of C-S due to the increase in the dipole moment. Therefore, the mechanical deformation changes the chemical environment in PEDOT, observed as the bonding and binding energy shifts of PEDOT induced by the local rearrangement of PEDOT into the PSS shells.

**Figure 4:**
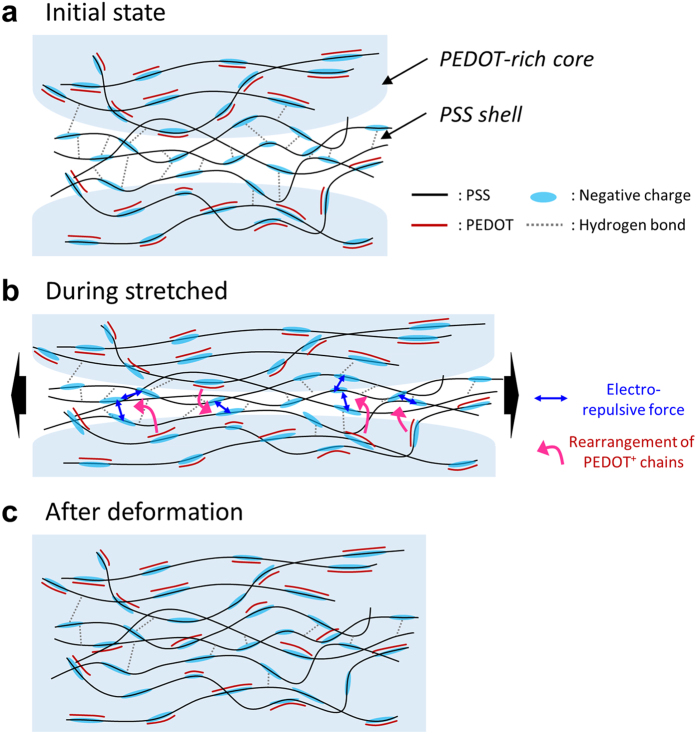
Schematic illustration of the evolution procedure of PEDOT-rich core by mechanical tensile stretch. During stretched, a local rearrangement of PEDOT chains occurred and the PEDOT chains locally migrated to the vicinity of PSS shell to reduce electro-repulsive force among PSS chains. The area of PEDOT-rich core increased after the tensile deformation.

Based on the above observations, the growth of PEDOT-rich cores can be explained. The PEDOT:PSS is primarily composed of two parts, a phase rich in PEDOT segments and a phase rich in excess PSS, which form the morphology of the PEDOT-rich core and PSS shell shown in Fig. 4. In the core region, the positively charged PEDOT segments are bonded with the negatively charged PSS chains through Coulomb interactions. The excess PSS chains are bonded to each other through hydrogen bonding, which forms the shells. These electronic forces stably maintain the PEDOT:PSS polymeric structure. When we mechanically stretch the PEDOT:PSS films, the electronic interactions among the PEDOT and PSS chains are significantly influenced by a large distortion of the polymeric structure. As shown in Fig. 4, as the films are stretched, the inter-distance between the PEDOT-rich cores gradually closes, and the thickness of the PSS shells thins. Hence, shorter the inter-distances result in stronger electric repulsion among the negatively charged PSS chains. The resultant repulsion leads to an electrical and structural instability between the cores. To relieve the electric repulsion, positively charged PEDOT segments locally rearrange and migrate to the vicinity of the PSS shells. Due to the rearrangement of the PEDOT segments into the shells, the PSS boundaries between the cores gradually disappear and become PEDOT segments bonded to PSS chains. Therefore, after the tensile deformation, the PEDOT-rich cores initially divided into two are agglomerated into one through coalescence with each other (Fig. 4). The increase in core area is induced by the local rearrangement of PEDOT segments into PSS shells, which leads to the growth of the conductive region and enhancement of charge transport.

### Dependence of the change in resistivity on the weight ratio of PEDOT to PSS

**Figure 5:**
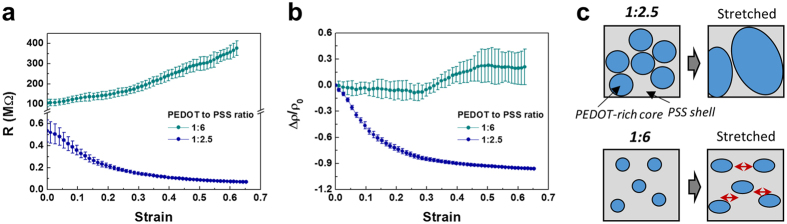
Opposite change in the PEDOT:PSS resistivity dependent on the weight ratio of PEDOT to PSS. (**a**) Resistance change of PEDOT:PSS films with tensile strain for the PEDOT to PSS ratio of 1:6 and 1:2.5. (**b**) Relative change in the resistivity as a function of the strain for the 1:6 and 1:2.5 weight ratio. (**c**) Schematic illustration of evolution of PEDOT-rich cores with the strain dependent on the weight ratio of PEDOT to PSS.

The decrease in electrical resistance of PEDOT:PSS under tensile strain is mainly caused by the evolution of conductive PEDOT-rich cores through the local rearrangement of PEDOT segments into PSS shells. The rearrangement with strain is critically influenced by the initial morphology of PEDOT:PSS. To investigate the effects of the PEDOT:PSS morphology on the resistance change behaviors, we prepared two types of PEDOT:PSS films with different weight ratios of PEDOT to PSS of 1:2.5 and 1:6. The relative weight of PSS increased for the PEDOT:PSS (1:6) compared with PEDOT:PSS (1:2.5). The increase in the PSS ratio definitely influences the change in the electrical properties according to the strain. Here, we measured the resistance change for the PEDOT:PSS films with ratios of 1:2.5 and 1:6 under stretched rupture strain (65% stain). As shown in Fig. 5a, large differences can be observed for the resistance change of the two films. First, the 1:6-PEDOT:PSS film has a much higher initial electrical resistance than that of the 1:2.5-PEDOT:PSS film. The initial resistances are 104 MΩ and 0.534 MΩ for the 1:6 and 1:2.5 films, respectively, because of the large increase in PSS weight in the PEDOT:PSS films. The excess PSS acts as an insulated barrier with a shell shape and hinders the charge transport on the films. Hence, a large increase in the initial resistance is observed due to the increase of the PSS weight ratio to PEDOT.

Interestingly, the opposite change in resistance with tensile strain was observed according to the increase in the PSS-to-PEDOT ratio. For the 1:6-PEDOT:PSS films, the resistance gradually increased as the applied strain increased. With an increasing PSS ratio, the resistance response with increasing strain is completely different from the previously observed decrease. We can infer that the large increase in the PSS ratio in the PEDOT:PSS films critically influences the change in resistance. To confirm the opposite behaviors more precisely, we replotted the resistance change with the relative change in resistivity as a function of strain, as shown in Fig. 5b. The previous equation (1) was used for the conversion. For the conventional 1:2.5-PEDOT:PSS films, the relative change in resistivity gradually decreased with increasing strain. However, the resistivity change of the 1:6-PEDOT:PSS films was almost invariant up to a low strain of 30% and then steadily increased with the strain of the films. This change in resistivity was completely different from that of the 1:2.5-PEDOT:PSS films, indicating that the growth of the conductive region due to the coalescence of PEDOT-rich cores does not occur with the increasing tensile strain. The morphology changes in such a way to increase the resistivity according to the tensile deformation.

In Fig. 5c, the morphological change of PEDOT:PSS is illustrated for PEDOT:PSS weight ratios of 1:2.5 and 1:6 upon stretching. In 1:2.5-PEDOT:PSS films, the PEDOT-rich cores coalescence with each other into larger cores with increasing strain on the films, leading to increased conductive regions and enhanced electrical conductivity. Conversely, for the 1:6-PEDOT:PSS films, the PEDOT-rich cores are relatively small, and the inter-distance between the cores is long due to the high weight of PSS in the films. Even after applying a large strain on the films, the cores could not coalesce. As the strain on the films increased, the cores elongated in the direction of the stretching, and the inter-distance between the cores spontaneously and gradually lengthened, leading to an increase in insulating PSS barriers and preventing the transport of mobile charges. Therefore, the electrical response is critically influenced by the initial morphology of the PEDOT:PSS films, which exhibit an opposite change in resistance with the strain according to the weight ratio of PEDOT to PSS.

## Discussion

The evolution of PEDOT-rich cores was induced by mechanical tensile deformation and exhibited overall core growth due to the consumption of smaller cores by larger cores. The resultant growth is caused by a local rearrangement of PEDOT segments into PSS shells to lower the electric repulsive forces between the negatively charged PSS chains in the vicinity of the shells. A transition of electron binding and bonding energy was also observed from the change in the chemical environment of PEDOT:PSS, induced only by the mechanical strain. With an increase in the conductive PEDOT-rich cores in the films, the electrical resistivity gradually decreased according to the strain due to the large increase in the conductive regions. Furthermore, the decreasing resistivity followed the same path as the strain, independent of the thickness of the PEDOT:PSS films, indicating that the growth mechanisms act equally on PEDOT:PSS, regardless of the thickness. When weight ratio of PSS to PEDOT was increased (1:6-PEDOT:PSS), completely different behaviors were observed in terms of the increase in resistivity with the tensile strain because of the enlarged insulating PSS barriers resulting from the increased PSS ratio in the PEDOT:PSS films. Therefore, the mechanical deformation of PEDOT:PSS directly affects the internal morphological change, and the resultant change leads to the transition in the electrical properties and chemical environment of PEDOT:PSS. An in-depth understanding of the change in strain-dependent resistivity will enable wide applications in strain sensors, actuators and the conductivity modulation tools to improve the performance of stretchable devices.

## Methods

### Preparation of PEDOT:PSS Solution and Films

PEDOT:PSS (Clevios^TM^ PH 1000 and P VP Al 4083 Heraeus, Germany) solutions were prepared and then filtered through polypropylene filters (0.45-μm pore size). The PEDOT:PSS (PH 1000) films were formed by spin-coating at 1000, 2000, and 3000 rpm for 30 s onto polyimide (PI) substrates (Kapton 300 HN, DuPont^TM^) treated with air plasma (plasma cleaner PDC-32G, Harrick Plasma). The PEDOT:PSS (P VP Al 4083) films were also formed by spin-coating at 1000 rpm for 30 s on a PI substrate. The spin-coated films were annealed on a hot plate for 15 min at 130 °C in an ambient N_2_ glove box and then cooled to room temperature in the glove box. Subsequently, the PEDOT:PSS films cast on the plasma-treated PI substrates were cut into rectangles measuring 10 mm in width and 30 mm in length.

### Characterization

Uniaxial stretching tests were conducted on the PEDOT:PSS films using a micro tensile machine (MMT-500N, Shimadzu) equipped with an electrically contactable jig for *in-situ* resistance measurements. All the tests were conducted at room temperature at a constant strain rate of 5 × 10^−5^ s^−1^. As the films were deformed by uniaxial tension, the electrical resistance of the films was measured with an Agilent 34410A multimeter. The initial electrical conductivities of the films were measured using the four-point probe technique. Surface images of the PEDOT:PSS films were acquired using an optical microscope and scanning electron microscopy (SEM, FE-SEM S-4800, Hitachi) at each strain. Atomic force microscopy (AFM) phase images were obtained with a NANO Station II instrument operating in non-contact tapping mode under ambient conditions. An X-ray photoelectron spectroscopy (XPS) survey spectrum was obtained using a PHI 5000 VersaProbe^TM^ (ULVAC-PHI) with a monochromatized Al Kα, 1486.6 eV source. Fourier transform infrared (FT-IR) spectroscopy measurements were recorded on a Vertex 80 spectrometer (Bruker) in absorption mode.

## Additional Information

**How to cite this article**: Lee, Y.-Y. *et al.* Growth Mechanism of Strain-Dependent Morphological Change in PEDOT:PSS Films. *Sci. Rep.* **6**, 25332; doi: 10.1038/srep25332 (2016).

## Supplementary Material

Supplementary Information
