# Supplementary material for: Growth Mechanism of Strain-Dependent Morphological Change in PEDOT:PSS Films
Source: Sci Rep. 2016 Apr 29;6:25332. doi: 10.1038/srep25332 (PMC4850459; doi:10.1038/srep25332)
Supplement: Supplementary Information [file srep25332-s1.pdf]

Growth Mechanism of Strain-Dependent Morphological Change in PEDOT:PSS Films

Yoo-Yong Lee[1], Gwang Mook Choi[1], Seung-Min Lim[1], Ju-Young Cho[3], In-Suk Choi[2], Ki Tae Nam[1*], and Young-Chang Joo[1*]

[1]*Department of Materials Science and Engineering, Seoul National University, Seoul 151-744, Korea, Republic of Korea*

[2]*High Temperature Energy Materials Research Center,Korea Institute of Science and Technology (KIST), Seoul, 136-791, Republic of Korea*

[3]*Institute of Physics (IA), RWTH Aachen University, Aachen, 52056, Germany*

*Corresponding authors:

Ki Tae Nam and Young-Chang Joo (E-mail: nkitae@snu.ac.kr, ycjoo@snu.ac.kr)

1. Resistivity change of PEDOT:PSS films on PI substrates

The change in the electrical resistance of the PEDOT:PSS films on PI substrates with increasing tensile strain was investigated using the *in-situ* resistance measurement tensile test system. The strain of the samples was monitored by optically measuring the distance between markers on the samples. Here, the measured resistance changes affect the resistance changes induced by the geometrical shape change of the films under tensile deformation. When a substrate is stretched in the longitudinal direction, the length (*l*) of the supported film increases, but the width (*w*) and thickness (*t*) decrease. Given that the resistivity (ρ) is constant under tensile deformation, the variation in resistance ($\Delta R_G/R_0$) induced by the geometric change can be described by equation (1):

$$\frac{\Delta R_G}{R_0} = \frac{R_G - R_0}{R_0} = \left[\frac{l/l_0}{(w/w_0)(t/t_0)} - 1\right] \quad (1)$$

where l/l_0 is the extension ratio of the film and w/w_0 and t/t_0 denote the contraction ratio of the width and thickness, respectively. The extension in length and contraction in width were optically measured by monitoring a marker's movement on the substrates. The contraction in thickness can be calculated, assuming that the film undergoes Poisson compression, $(1-\nu_f\varepsilon_l)$, where ν_f is the Poisson's ratio of PEDOT:PSS (0.35) and ε_l is the longitudinal strain. Using both the measured resistance change and the change induced by the geometrical shape change, the resistivity can be obtained. To determine the resistivity change, the geometric contribution was calculated and subtracted from the measured resistance change. The relationship between the relative resistance and the resistivity change can be expressed by equation (2):

$$\frac{R}{R_0} = \frac{\Delta R}{R_0} + 1 = \frac{\rho}{\rho_0} \cdot \left[\frac{l/l_0}{(w/w_0)(1-\nu_f\varepsilon_l)}\right] = \frac{\rho}{\rho_0} \cdot \left[\frac{\Delta R_G}{R_0} + 1\right] \quad (2)$$

where $\Delta R/R_0$ and $\Delta R_G/R_0$ are the normalized change in the measured resistance and that induced by the change in geometry, respectively. ρ_0 is the initial resistivity. The equation can be rearranged to obtain the resistivity of the PEDOT:PSS films at each strain level:

$$\rho = \rho_0 \cdot \left[\frac{\Delta R}{R_0} + 1\right] \Big/ \left[\frac{\Delta R_G}{R_0} + 1\right] \quad (3)$$

2. Resistivity change at the direction perpendicular and parallel to the tensile direction

We have measured the resistivity changes at the direction not only parallel but also perpendicular to the tensile direction as shown in Figure S2. During the mechanical deformation, the resistivity decreased at the parallel diection but increased along the perpendicular direction below the strain of 7%. However, under large mechancial strain, the resistivity at the direction perpendicular to the tensile direction is also decreased with the strain. The both resistivity changes decreased with the strain which have similar slope of the change. It is mainly caused by the equivalent growth of the conductive PEDOT-rich cores independent of the tensile direction (Figure 2a). The electrical conducuction is enhanced in the both direction due to the coalescence of the cores induced by large mechancial strain.

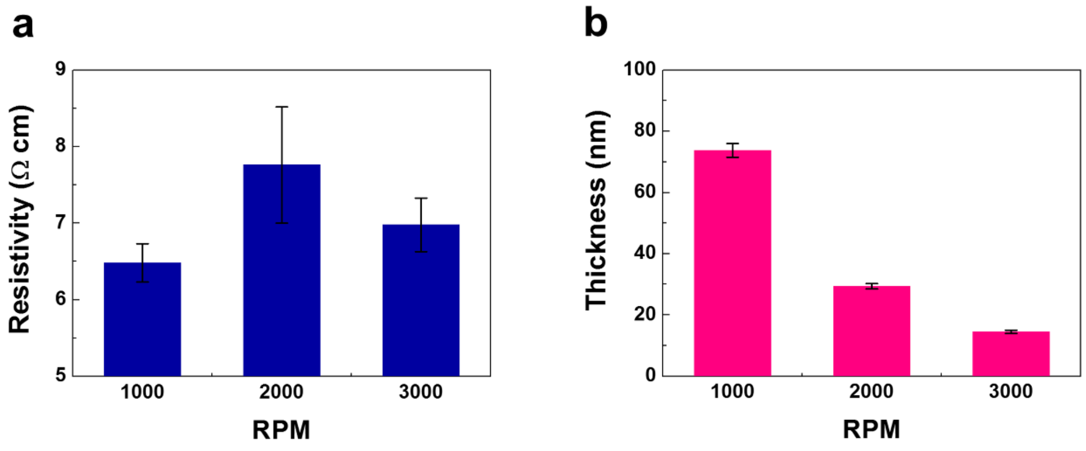

Figure S1. (a) Electrical resistivity of PEDOT:PSS films as a function of the rpm of the spin-coater. (b) Thickness of PEDOT:PSS films as a function of the rpm of the spin-coater.

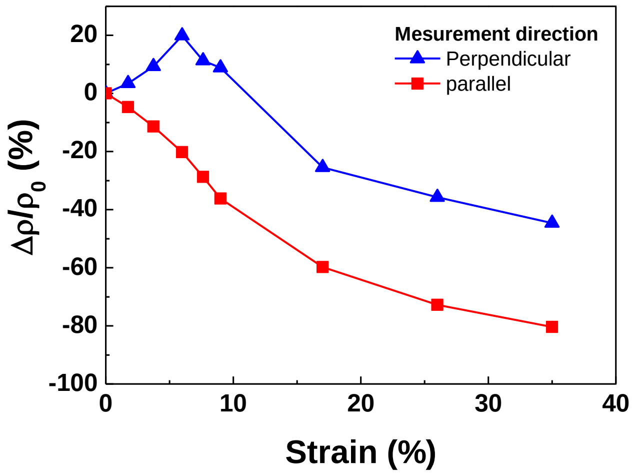

Figure S2. Electrical resistivity changes of PEDOT:PSS film at the direction perpendicular and parallel to the tensile direction.

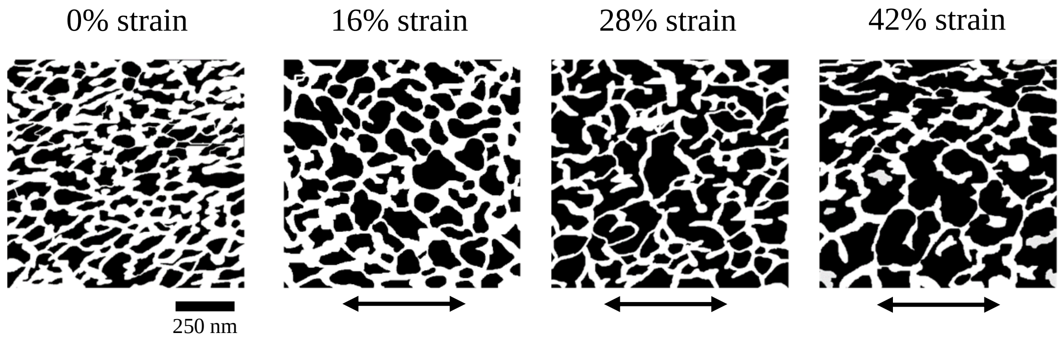

Figure S3. AFM phase images of PEDOT:PSS films at each strain reproduced using image analyzing software (Scion Image analyzer). Black and white areas in the images represent the PEDOT-rich cores and PSS shells, respectively. The arrows indicate stretching direction.

Table S1. Representative FT-IR absorption band for PEDOT:PSS and PI films.

**PEDOT:PSS**	**Wave # (cm^{-1})**	**PI**	**Wave # (cm^{-1})**
C-S (PEDOT)	709	C=O bending	723
	862		
	944		
C-C (PEDOT)	1288	C-O-C	1086
C=C (PEDOT)	1496	C-N	1377
S-O (PSS)	1122	C=C	1600
	1162	C=O	1714
S-phenyl bonds (PSS)	1037		1775
